# Evolution of ischemic damage and behavioural deficit over 6 months after MCAo in the rat: Selecting the optimal outcomes and statistical power for multi-centre preclinical trials

**DOI:** 10.1371/journal.pone.0171688

**Published:** 2017-02-09

**Authors:** Sarah S. J. Rewell, Leonid Churilov, T. Kate Sidon, Elena Aleksoska, Susan F. Cox, Malcolm R. Macleod, David W. Howells

**Affiliations:** 1 Florey Institute of Neuroscience and Mental Health, Melbourne Brain Centre, Austin Campus, Heidelberg, Australia; 2 Department of Clinical Neurosciences, University of Edinburgh, Edinburgh, United Kingdom; 3 University of Tasmania, School of Medicine, Faculty of Health, Hobart, Australia; University of Münster, GERMANY

## Abstract

Key disparities between the timing and methods of assessment in animal stroke studies and clinical trial may be part of the reason for the failure to translate promising findings. This study investigates the development of ischemic damage after thread occlusion MCAo in the rat, using histological and behavioural outcomes. Using the adhesive removal test we investigate the longevity of behavioural deficit after ischemic stroke in rats, and examine the practicality of using such measures as the primary outcome for future studies. Ischemic stroke was induced in 132 Spontaneously Hypertensive Rats which were assessed for behavioural and histological deficits at 1, 3, 7, 14, 21, 28 days, 12 and 24 weeks (n>11 per timepoint). The basic behavioural score confirmed induction of stroke, with deficits specific to stroke animals. Within 7 days, these deficits resolved in 50% of animals. The adhesive removal test revealed contralateral neglect for up to 6 months following stroke. Sample size calculations to facilitate the use of this test as the primary experimental outcome resulted in cohort sizes much larger than are the norm for experimental studies. Histological damage progressed from a necrotic infarct to a hypercellular area that cleared to leave a fluid filled cavity. Whilst absolute volume of damage changed over time, when corrected for changes in hemispheric volume, an equivalent area of damage was lost at all timepoints. Using behavioural measures at chronic timepoints presents significant challenges to the basic science community in terms of the large number of animals required and the practicalities associated with this. Multicentre preclinical randomised controlled trials as advocated by the MultiPART consortium may be the only practical way to deal with this issue.

## Introduction

Animal models have been widely used to aid our understanding of stroke pathophysiology and the potential effectiveness of putative therapies. However, their utility has been questioned because of the failure to translate positive animal model data to a successful clinical treatment. At the core of this translational failure lie key differences in the endpoint and outcome measures between experimental and clinical studies. Assessment of neurological deficit and disability in patients months after stroke is in stark contrast to many experimental animal studies where infarct volume assessed at acute timepoints after stroke is the main outcome reported [1–6]. This disparity in what and when we measure prompted the STAIR recommendations that pre-clinical studies extend monitoring to at least 30 days, and include multiple outcome measures including both behavioural testing and infarct volume assessment [7, 8]. However, despite being introduced over 15 years ago, these recommendations have yet to make a great impact on translation of an acute stroke therapy [6, 9–11]. Indeed, of over 3000 experiments collected by the CAMARADES group for systematic review, only 5% assessed outcome at times beyond one month, with 85% assessing outcome within one week of stroke (CAMARADES, data on file).

Meeting these challenges and facilitating rigorous evaluation of candidate therapies requires a clear understanding of methodological variability and how experiments need to be powered to reliably quantify outcome. Chronic endpoints in animal models are also made challenging by survival, paucity of detectable and long lasting behavioural deficits, and consistency in infarct size [12].

To this end, improvements to each animal model of stroke have been made to result in more consistent infarct sizes and improved survival. For example through different thread properties and occlusion durations in the thread occlusion model; manipulation of photosensitive dye concentration, illumination period or laser settings in the photothrombotic model; and location and length of artery occluded in direct occlusion models [13–16], infarcts of different size and location can be induced with relative consistency. While reducing variability makes modelling cheaper, it does so at the expense of generalizability. Human stroke is also highly variable.

This study aims to profile neurological deficit and histological damage in cohorts of rats collected at varying times up to 24 weeks following transient thread occlusion stroke. In addition, we aim to examine variability in outcomes measured and the impact on sample size calculations for future studies. By using a multi-cohort design allowing us to combine behavioural measures with assessment of histology at late timepoints after stroke, we hope to better align experimental stroke models with the clinical situation.

## Materials and methods

### Animals & ethics

All procedures involving animals were approved by the Animal Ethics Committee of Austin Health (Heidelberg, Victoria, Australia, approval number 2007/2796) and performed in accordance with institutional, national and the Animal Research: Reporting *In* Vivo Experiments (ARRIVE) guidelines (Australian code of practice for the care and use of animals for scientific purposes, 7^th^ edition, 2004)[17]. Male Spontaneously Hypertensive Rats (SHR) were used throughout this experiment (Animal Resource Centre, Canning Vale, Western Australia). All animals were aged 15 weeks at surgery (286-362g). Blood pressure was not monitored.

### Sample size calculation

Based on previous infarct volume data using an identical surgical design (90 minute thread occlusion MCAo in 15 week old male SHR), a sample size calculation (power = 0.8; alpha = 0.05, see below) estimated 11 animals per group would be required to detect an infarct equivalent to 25% of the uninjured hemisphere. Additional animals were allocated to longer recovery timepoints to allow for deaths and to ensure enough animals survived to each time point. A total of 132 animals underwent surgery for this experiment.

### Randomisation and blinding regime

To reduce selection, performance, detection and attrition biases, care was taken to ensure animals were randomised to experimental group, investigators remained blinded to experimental group for induction and assessment of ischemia, and outcomes for all animals used in the experiment were reported. Specifically, animals were numbered upon arrival at the animal facility. An investigator independent of the surgical procedure randomised the list of animals to experimental group (using Excel random number generator), with surgical type (stroke or sham) being revealed to the surgeon immediately prior to thread insertion. Animals undergoing ischemia were further randomised to one of nine experimental groups that determined when they were to be collected for histology analysis: 1, 3, 7, 14, 21, 28, 84, and 168 days following MCAo. Sham animals (complete surgery without thread insertion) were sacrificed for histology at 168 days following surgery. In the case where an animal died prior to its designated endpoint, the animal was replaced from a pool of additional animals. Data from animals which did not reach their designated endpoint was not included in the analysis except for a summary of mortality. Allocation to group remained blinded throughout the behavioural assessment, with animals carrying no identifier of experimental group. Once animals reached their designated endpoint, tissue was collected and re-coded to ensure blinded histology. All surgery, behavioural testing and histological analysis were performed by a single investigator.

### Induction of focal ischemia

Anaesthesia was induced by inhalation of 5% isoflurane (mixed with 50:50 air and oxygen) in an enclosed box and then maintained using a nose cone where 2% isoflurane (mixed with 50:50 air and oxygen) was delivered to the spontaneously breathing animal for the duration of surgery. Throughout surgery, body temperature was monitored and controlled to 37.4°C using a rectal temperature probe controlling a heat mat (manufactured in house).

Focal cerebral ischemia was induced using the thread occlusion model of MCAo following the methods of Longa with modifications by Spratt [16, 18]. Cerebral blood flow was monitored in the area 5mm lateral, 1mm posterior to bregma by Laser Doppler flowmetry (MoorLab, Devon, UK, coupled to iWORX, Dover, NH, USA). Prior to affixing the Laser Doppler probe, the skull was thinned using a hand held drill. The right MCA was occluded for 90 minutes before withdrawal of the thread to allow reperfusion for the remainder of the experiment (24 hours to 24 weeks). Briefly, a silicone coated 4–0 nylon suture of dimensions 0.35mm diameter; 2mm coating length (manufactured according to Spratt et al[16]) was inserted approximately 18mm through a stump created from the External Carotid Artery to occlude the MCA until mild resistance was felt. This coincided with a drop in blood flow. Animals were recovered from anaesthesia following occlusion. Just prior to 90 minutes post MCAo, animals were reanaesthetised to permit withdrawal of the thread and reperfusion. Animals allocated to the sham group underwent the same surgical procedure as stroke groups, without insertion of the occluding thread. This included clips being briefly placed on the ICA and CCA.

All animals were housed individually following surgery to ensure a consistent post-surgical environment. Postoperative analgesia was provided in the form of a single rectal suppository of paracetamol (5mg of a 20mg/kg solution). A post-operative care plan was employed to help prevent weight loss and dehydration over the first week after surgery. This included the supply of paracetamol in drinking water (120mg/kg), sub-cutaneous injections of saline (days 1–3) and provision of soft food. For the first week after surgery, animals were housed in a recovery room where they could be closely monitored. After the seventh day post-surgery, animals were returned to live in general holding rooms, still in individual cages. Weight, basic behaviour and general health were monitored daily for the first two weeks following surgery, and regularly thereafter.

### Behavioural tests

Three measures were used to assess behavioural deficit after stroke: basic behavioural scale, adhesive removal test, and sunflower seed challenge. All three behavioural assessments were performed at the following times: Pre, 1, 3, 7, 14, 21, 28 days, 8, 12, 16, 20, 24 weeks.

#### Basic behavioural scale

**Fig 1:**
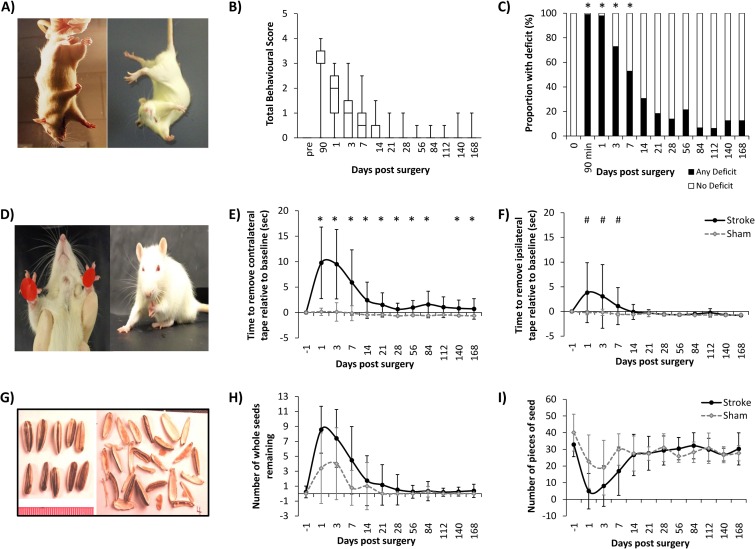
Assessment of behavioural deficit for 6 months after stroke. Basic Behavioural Test: A) Forelimb flexion and torso twisting. B) Total score over the timecourse of the experiment. C) Presence of a deficit (shaded) over the course of the experiment. Adhesive Removal Test: D) Placement and removal of tape on the forepaws. E) Time to remove contralateral (left) tape relative to baseline ability. F) Time to remove ipsilateral tape relative to baseline ability. Sunflower Seed Challenge: G) Intact and broken seeds. H) Number of whole seeds remaining. I) Number of pieces of seed.

Neurobehavioural deficit was assessed using a modified version of the methods described by Petullo et al to score forelimb flexion, torso twisting, lateral push resistance, and general mobility (Table 1, Fig 1A)[19]. This test was chosen as a quick and simple assessment of neurological deficit after stroke.

**Table 1:** Scoring criteria for Basic Behavioural Test after stroke (Modified from Petullo et al)[19].

Test	Score	Description
Forelimb Flexion	0	**No flexion.** Forelimbs extend equally outstretched toward the bench
	0.5	**Mild.** Left forelimb at approximately 45° angle consistently; or angle is closer to 90° but is not consistent.
	1	**Moderate to severe.** Left forelimb is at 90° or greater. Forelimb flexion is consistent.
Torso Twisting	0	**No signs of body rotation.** Body elongated and extended toward the bench.
	0.5	**Mild.** Half twist of the body.
	1	**Moderate to severe.** Consistently strong twisting to contralateral side. Head and forelimbs brought toward hind limbs.
Lateral Push	0	**Equal resistance.** Animal resists being pushed.
	0.5	**Weakened resistance.** Animal shows weakened resistance whilst trying to correct.
	1	**No resistance.** Animal has severely weakened resistance. Tends to “roll” with left legs collapsing after being pushed to the left.
Mobility	0	**Normal mobility.** Animal is able to freely walk, move about cage, rear on hindlimbs.
	0.5	**Spontaneous movement reduced.**
	1	**Needs stimulus to move.**
	2	**Unable to walk.**

#### Adhesive removal test

The adhesive removal test (also referred to as the sticky tape test) has been used as a measure of motor co-ordination and sensory neglect after stroke in experimental studies using rats and mice [20–27]. After a period of familiarisation in the glass test box (30x28x45cm), the rat was lightly restrained to allow attachment of 9mm circular office stickers (Checkpoint Systems, Kuala Lumpur, Malaysia) to the palm of each forepaw (Fig 1D). The order of attachment (left or right applied first) was alternated at each test. Additionally, each forepaw was touched firmly and in quick succession after attachment of the tape. The time taken to remove each sticker was recorded. Three trials were undertaken in each test session, with a rest period of at least 1 minute between trials. A maximum time of 150 seconds was allocated for each trial. If the stickers could not be removed within this period, a maximum time was recorded. Animals were acclimatized to the test procedure in the week prior to surgery via 3 training sessions of 3 tests. A baseline test was recorded on the day prior to surgery.

#### Sunflower seed challenge

The fine motor co-ordination required to manipulate, open and consume a sunflower seed from its husk may provide an opportunity to explore long term deficit following stroke [28, 29]. To simplify the test, we examined the number of pieces of sunflower seed remaining after a set time. Rats were given sunflower seeds in their home cage to familiarise them with shelling and consuming seeds throughout the week prior to surgery. At each test session, rats were placed in a paper lined box with 10 sunflower seeds. After 30 minutes the number of whole (uneaten) seeds and pieces of shell were recorded (Fig 1G). Animals were not fasted prior to testing.

### Tissue collection and processing

At their designated endpoint, animals were deeply anaesthetised with isoflurane and transcardially perfused with 90mL normal saline followed by 270mL 4% paraformaldehyde. The brain was removed and postfixed in paraformaldehyde for 24 hours before being cut into 2mm thick coronal slices which were then processed and embedded in paraffin wax. Seven micron sections were collected on to silane coated microscope slides, followed by staining with hematoxylin and eosin for infarct delineation.

### Infarct analysis

#### Image acquisition

Slides were digitised using an Aperio ScanScope XT microscope (Leica Biosystems, Wetzlar, Germany) at 20x magnification. The pen tool within ImageScope (Aperio Technologies, Vista, CA, USA) was used to manually define hemispheres and ventricles, and to delineate areas of ischemic injury (described below).

#### Definitions of damage

Progression of ischemic related damage involves many stages which influence the structure and appearance of tissue. Normal healthy tissue was characterised by round neuronal nuclei and cell bodies, with uniform neuropil staining. The absence of normal cells was used to define the boundary for outlining ischemic related damage. As the infarct aged and progressed, differentiation between striatal and cortical damage became more difficult and so we report the total area damaged. Qualitative notes were made of the location of damage.

Areas of damaged tissue were delineated based on descriptions provided in the literature [30–33]. Vacuolation and sponginess of the neuropil, pallor of the eosiniphilic background and the shape and staining of neuronal cell bodies were used to identify acutely damaged tissue [31]. Early ischemic damage included pyknosis where cells appeared shrunken and intensely stained, and cells with eosinophilic cytoplasm (red neurons), together with disruption of the neuropil. Neuronal damage progressed to ghost neurons where affinity for hematoxylin was lost, before fading into the neuropil [34]. Inflammatory cells invaded the area of damage and could be identified by their small dark round nucleus. These cells are likely to be neutrophils [35–37]. Macrophages also entered the ischemic area, characterised as large round cells with an offset nucleus. As the ischemic area was cleared by phagocytosis, a dense band of glial cells (with small nuclei and mesh like morphology) formed a border between healthy tissue and cavity. This band was included in measurements of damaged tissue.

#### Calculation of infarct volume

Volume of damage was calculated based on six slices approximately 2mm apart, ranging from 3.7 to -6.3mm relative to bregma. Area measurements were converted to volumes by finding the average area at two adjacent planes and multiplying by the distance between the planes. Volumes were calculated for each hemisphere, total brain size, ventricles, damaged tissue, and healthy tissue (remaining ipsilateral tissue). To account for differences in hemisphere size due to the effects of oedema and atrophy, total ischemic damage was calculated as a proportion of the contralateral hemisphere using the formula $\frac{\left(contralateral\;area-remaining\;ipsilateral\;area\right)}{contralateral\;area}$ [24].

### Statistics and analysis

Statistical analyses designed by our statistician (LC) were conducted using IBM SPSS Statistics v20 and Stata 13IC (StataCorp). Groups were compared using ANOVA with Bonferroni post hoc analysis for between group comparisons; and Dunnetts test for comparison to sham. When there was significant variation between experimental groups (failure of Levene’s test of homogeneity of variances), Game-Howell and Dunnett T3 post hoc tests were applied. To test for differences in body weight over time (comparing stroke and sham animals), random-effect generalised least squares regression was used. Linear regression was used to compare individual timepoints. Cox regression with shared frailty, adjusting for time and baseline ability was used to examine the adhesive removal test. Data for the basic behavioural test was recoded in a binary fashion (deficit / no deficit) and examined using random effects logistic regression. Cross tabs, Fisher’s exact test and Agresti’s generalised odds were used to examine deficit at individual timepoints [38]. Sample size and power calculations were performed using an online calculator (http://www.statisticalsolutions.net/pssTtest_calc.php), power = 0.8, α = 0.05. All infarct data are presented as average ± standard deviation, with individual animal data points displayed on graphs. The degree of association between deficit in the adhesive removal test assessed at day 1 and ischemic damage was estimated with Pearson correlation coefficient.

## Results

### Summary of animal numbers

A total of 132 animals underwent surgery. Twenty-eight animals died prematurely or were excluded from the study. Most deaths (n = 26) occurred within 21 days of stroke. Of these, 7 animals died within a day of surgery due to sub-arachnoid haemorrhage or technical error during stroke induction. Potential infection was suspected in 7 animals, characterised by blood clots lining the chest cavity [39, 40].

Importantly, all animals who reached their designated endpoint showed both behavioural and histological evidence of successful stroke. Of 104 animals who reached their endpoint, 98 were available for histological analysis; 6 brains were damaged during tissue processing (Table 2). For behavioural tests, data from stroke animals was grouped and compared to sham for each assessment timepoint. The number of animals tested at each time is summarised in Table 2.

**Table 2:** Summary of cohort sizes, CBF drop at MCAo and Tissue Damage (Mean±Standard Deviation).

		24 hours	3 days	7 days	14 days	21 days	28 days	12 weeks	24 weeks	Sham
**n**	**Behavioural Assessment**	93	83	73	62	51	40	29	15	9
	**Reached Endpoint**	11	11	11	11	11	11	13	16	9
	**Infarct Analysis**	11	11	11	10	11	11	12	15	6
**CBF decrement at occlusion (% of baseline)**		73.3±9.9	65.5±14.0	66.3±17.6	73.5±8.0	70.4±15.6	74.4±11.0	66.7±19.1	73.0±17.0	-24.3±24.3
**Reperfusion Recovery (% of pre MCAo)**		74.5±77.4	113.6±81.7	127.6±150.7	109.3±144.1	312.4±382.7	160.7±269.9	120.6±121.7	285.0±692.3	125.2±103.0
**Volume of Damage (mm^3^)**		134.4±50.1	142.2±43.4	93.7±33.5	88.9±29.1	93.7±27.8	80.9±20.6	50.1±23.5	60.7±24.7	4.5±9.9
**Total Ischemic Damage (%)**		28.5±9.8	29.5±9.0	26.0±6.9	34.4±12.0	33.0±9.8	32.8±7.8	26.3±9.0	29.1±12.6	5.1±5.2

### Confirmation of successful stroke induction

Induction of MCAo was confirmed by a drop in blood flow at insertion of the occluding thread and acute behavioural deficits. The average CBF decrement at MCAo relative to pre-occlusion was 70.6±14.6%. The degree of occlusion was equivalent across all stroke groups, and significantly different to sham (F(8,91) = 40.43, p<0.001) (Table 2).

At 90 minutes (immediately prior to reperfusion) all stroke groups had a median behavioural score of 3, with scores ranging from 1.5–4, suggesting MCAo induced a similar degree of deficit in all stroke animals. Sham animals showed no deficit 90 minutes after surgery, suggesting the deficits seen in stroke animals are due to ischemia and are not caused by the surgical procedure or anaesthesia.

All animals exhibited reduction in Laser Doppler signal, behavioural deficit after withdrawal of anaesthesia and infarction at their designated endpoint. Therefore all procedures were considered successful.

### Behavioural deficit

#### Basic behavioural test

Deficit in the basic behavioural test was most pronounced at 90 minutes (immediately prior to reperfusion) (Fig 1B). Deficit was still evident but less severe at day 1 and continued to diminish over the first week. At timepoints beyond 14 days there appears to be very little behavioural deficit detected using the basic behavioural test.

Scores were re-coded to group animals with no deficit (total score = 0), or any deficit (total score >0.5) to allow testing of repeated assessments. Random effects logistic regression found a significant difference between stroke and sham groups across the entire experiment (Odds Ratio = 940.04; 95% CI: 61.64, 1436.5; p<0.001), with a significant treatment-by-time interaction. When individual timepoints were considered, stroke animals were more likely to have a deficit (any score) in the basic behavioural test than sham animals up to 7 days post-surgery (Odds Ratio = 3.24, 95% C.I 2.34, 4.49, p<0.001) (Fig 1C). At timepoints beyond 7 days, the basic behavioural test was not able to discriminate between stroke and sham animals as both groups had little or no deficit (14 day Odds Ratio = 1.88, 95% C.I. 1.46, 2.43, p = 0.063). Of the test components, forelimb flexion had the most longstanding (and strongest) deficit, but at later timepoints only a small proportion of animals (12.5%, n = 2) still had evidence of forelimb flexion.

#### Adhesive removal test

Prior to surgery, all animals were able to remove the tape rapidly (13±8 sec; 14±9 sec contralateral (left) and ipsilateral (right) forepaw respectively) and did not show preference in the order in which tapes were removed. Following induction of MCAo, pronounced deficit in their ability to remove the contralateral tape was observed (Fig 1E). This continued as an asymmetric bias to remove the ipsilateral tape before attempting to remove the contralateral tape for the entire observation period. Across all timepoints assessed, median time to remove the contralateral tape was 44±5 seconds (95% CI: 33.0, 51.67) for stroke animals, and 6±0 seconds (95% CI: 4.67, 6.33) for sham. Stroke animals were 94% less likely to remove the contralateral tape at any time over the 6 month experimental period compared to sham (Cox regression with shared fragility and adjustments for time and baseline ability. Hazard ratio = 0.063 (95% CI: 0.034, 0.115; p<0.001)). When individual timepoints were compared, stroke animals consistently removed the contralateral tape significantly slower than sham animals for all timepoints except 112 days (Fig 1E).

Impairments were also evident in the time to remove the ipsilateral tape (HR = 0.557; 95% CI: 0.339, 0.913; p = 0.020) (Fig 1F). When individual timepoints were examined, there were significant differences between stroke and sham animals in the time to remove the ipsilateral tape for the first week after stroke (Fig 1F). Beyond 7 days, there was no significant difference between stroke and sham animals, with each as likely to remove the ipsilateral tape.

#### Sunflower seed challenge

Prior to surgery, all animals could readily break all 10 sunflower seeds open, resulting in 34±8 pieces. Over the acute 3 days following surgery, many stroke and some sham animals did not open some (or all) sunflower seeds (Fig 1H). During the first 14 days, stroke animals broke sunflower seeds into fewer pieces than sham, leaving more seeds untouched (Fig 1I). After 14 days, stroke and sham animals broke sunflower seeds into an equivalent number of pieces. In its current form, this test does not appear useful for discriminating between stroke and sham animals, nor for assessing deficits after stroke.

### Evolution of ischemic related damage

**Fig 2:**
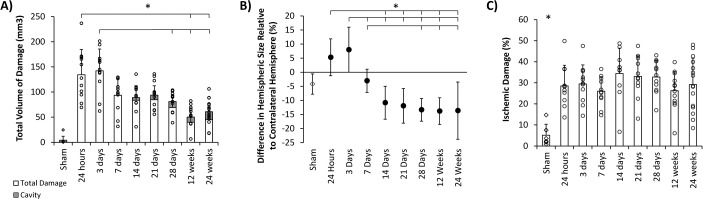
Ischemic damage over time after stroke. A) Total volume of damage including cavity (shaded). B) Changes in hemispheric size C) Ischemic damage corrected for the effect of oedema and atrophy. All values Mean±Standard Deviation. Animals are represented by individual data points. * p<0.05

Damage resulting from tMCAo developed rapidly, with an infarct encompassing the cortex and striatum evident by 24 hours. The volume of damage was largest at 3 days post stroke (142.2±43.4mm^3^) (Table 1, Fig 2A). From day 7 to 28 the volume of damage was smaller but stable, however the composition of this area of damage included larger volumes of cavity over time (Fig 2A). Cavity was first evident as small patches at 7 days, and became prominent in most animals by 14 days. The volume of cavity was largest at 24 weeks, representing 26.2% of the total damage (15.9±9.2mm^3^ cavity; 60.7±24.7mm^3^ total volume of damage). Infarct volume differed significantly over time (F(8,89) = 15.649, p<0.001), with all stroke groups significantly different to sham. Animals collected at 24 hours and 3 days had infarct volumes significantly larger than animals collected at 12 and 24 weeks (and 28 days for 3 day comparison) (p<0.05).

Changes in hemispheric size were evident early after stroke as an enlargement of the ipsilateral hemisphere relative to its contralateral counterpart (Fig 2B). Swelling of the ipsilateral hemisphere peaked at 3 days. By 7 days the effect of oedema had resolved, with ipsilateral and contralateral hemispheres being of a similar size, and comparable to sham. Beyond 14 days, the ipsilateral hemisphere was consistently smaller than the contralateral hemisphere, suggesting atrophy. The difference between ipsilateral and contralateral hemispheres differed significantly over time F(8,95) = 17.662, p<0.001. Only the 7 and 14 day groups did not differ from sham.

When the amount of tissue damaged was adjusted for differences in hemisphere size (due to the degree of atrophy or oedema), the proportion of ischemic damage was equivalent across all stroke groups (26.0±6.9%–34.4±12.0%) (Table 1, Fig 2C). One way ANOVA showed a significant difference in tissue loss between experimental groups (F(8, 89) = 5.592, p<0.001). All stroke groups had significantly greater tissue loss compared to sham (p<0.05). There was no difference in tissue loss between stroke groups collected at different timepoints, suggesting an equivalent volume of brain is damaged across all stroke animals after 90 minutes tMCAo in the SHR.

When the degree of deficit was compared with final ischemic damage, the best correlation was seen when comparing time to remove the contralateral tape in the adhesive removal test at day 1, when the deficit was most severe, with infarct volume measured at 24 weeks after stroke (r = 0.739, p = 0.003, n = 14).

#### Qualitative description of damage

Ninety minute transient MCAo in the SHR resulted in damage to the right striatum and cortex of all animals. Over time, more animals showed damage to the hypothalamus, hippocampus and thalamus (54% at 3 days, 54% at 21 days, 75% at 12 weeks respectively). Across all timepoints, scattered necrosis was noted in the contralateral hemisphere.

**Fig 3:**
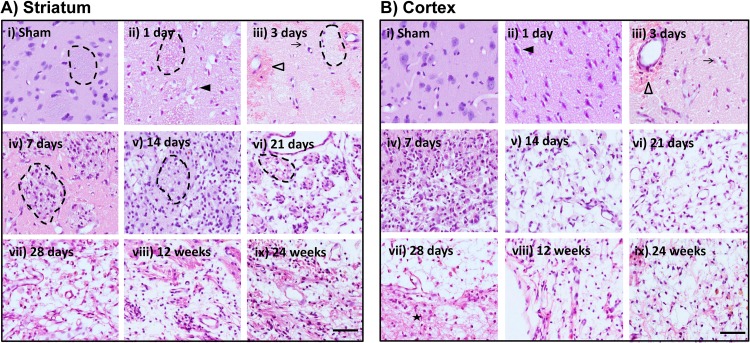
Evolution of ischemic related damage resulting from MCAo. Representative images taken through the centre of the area of damage at approximately 1.7mm relative to bregma. A) Striatum. B) Cortex. Scale bar represents 50μm. Filled arrow head = red neuron; Open arrow head = hemorrhagic transformation; Arrow = Ghost neuron; Dotted line = striatal bundle; Star = Glial scar.

As time progressed after stroke onset, tissue structure and the type and extent of damage differed. Sham animals generally showed normal histology on H&E stained sections, characterised by large neuronal nuclei and uniform neuropil. Early ischemic damage was typified by red neurons with shrunken pyknotic nuclei, eosinophilic cytoplasm and a “halo” where they had retracted from the surrounding neuropil (Fig 3A ii and 3B ii). The area of infarct showed vacuolation, sponginess, and a general sparseness of the neuropil. At 3 days, many neurons had taken on the ghost profile, fading into the background. Both striatum and cortex showed hemorrhagic transformation (Fig 3A iii and 3B iii). By 7 days the area of ischemic damage became hypercellular, packed with macrophages and other infiltrating cells. Within the damaged striatum, infiltrating cells were generally localised within the white matter bundles (Fig 3A iv). Clearance of ischemic tissue progressed through 14 and 21 days, with all animals showing growing areas of cavity amid a diffuse matrix of fine connective tissue at 21 days (Fig 3A vi and 3B v, vi). By 28 days a dense band of cells provided a border between damaged and intact tissue (Fig 3B vii). At 12 and 24 weeks, large portions of the ischemic area were a fluid filled cavity. Three animals showed mineral deposition within the thalamus.

### Sample size calculations

As the adhesive removal test showed persistent deficit in stroke animals for the entire experimental period, we calculated the sample size required to detect different degrees of recovery across the range of timepoints examined here (Table 3). When comparing to a sham group to detect the presence of a stroke, manageable cohort sizes of 9–36 (1 day and 24 weeks respectively) make the adhesive removal test a practical outcome measure. However, calculations to support differing degrees of recovery between stroke groups indicate that much larger numbers of animals would be required. For example, to detect a 25% improvement in behavioural deficit at 28 days, 842 animals per cohort would be required.

**Table 3:** Sample size calculations for behavioural and histological based outcome measures.

	Behaviour–Adhesive Removal Test					Ischemic Damage			
Time of assessment post stroke	Sham	10% recovery	25% recovery	50% recovery	75% recovery	10% difference	25% difference	50% difference	75% difference
**1 day**	9	809	130	33	15	186	30	8	4
**3 days**	9	810	130	33	15	147	24	6	3
**7 days**	18	1,847	296	74	33	111	18	5	2
**14 days**	25	3,417	547	137	61	192	31	8	4
**21 days**	24	3,952	633	159	71	139	23	6	3
**28 days**	13	5,262	842	211	94	89	15	4	2
**12 weeks**	23	4,284	686	172	77	184	30	8	4
**24 weeks**	36	12,581	2,013	504	224	295	48	12	6

When ischemic damage was considered the primary outcome, the required number of animals per cohort was smaller due to decreased variability (compared to the adhesive removal test) (Table 3). For the same example of 25% difference in ischemic damage assessed at 28 days, 15 animals per cohort would be required.

## Discussion

There are many challenges associated with bringing animal models of stroke in line with clinical studies, meeting the STAIR criteria and achieving this using a rigorous experimental design. These challenges include survival, long lasting and detectable behavioural deficits, and consistency in successful stroke induction and infarct size [12]. In this study, we aimed to explore long term outcomes in groups of rats after stroke, focussing on behavioural and histological endpoints.

### Survival

A key difference in outcome measures between clinical and experimental studies is the reporting of mortality. Outcome from a clinical treatment is often assessed by improvements in mortality [41], whereas mortality is rarely reported in the experimental literature, or included as part of outcome assessments [42–44]. Our animal models have been tailored to reduce mortality associated with the stroke and maximise survival, both for ethical and financial reasons. When it is reported, mortality following stroke in laboratory animals is in the order of 25% within 24 hours of stroke onset [45–50]. During the following days where oedema is maximal, mortality can increase to 33% [26, 51]. Nonetheless, honest reporting of animal numbers, including those that died and the reasons for it are important in taking animal models of stroke forward [44].

We have shown that moderately large infarct involving the MCA supplied cortex and striatum can be consistently induced in SHR rats with mortality (21%) broadly limited to two key periods–sub-arachnoid haemorrhage at thread insertion and development of suspected infection within 21 days. The ability to maintain animals for up to 6 months after stroke with limited mortality beyond the acute period is encouraging. This is entirely consistent with human data where mortality within 28 days is in the order of 28% [52, 53].

Whilst models that induce smaller infarcts such as photothrombosis can result in reduced mortality, they do not permit the study of reperfusion, and recovery of behavioural deficit is often rapid [54–56]. Long term studies involving mouse models of stroke have proven especially challenging, with mice being particularly susceptible to post-stroke infection [57] and inadequate food/water intake [58]. In this regard it is interesting that sham animals here exhibited difficulty consuming sunflower seeds during the acute post-surgical period, presumably because of ischemia to the muscles of the jaw [59]. These factors contribute to mortality in mouse stroke models being much higher than rats, up to 90% [60, 61].

### Detectable and long lasting behavioural deficits

The ability to detect and assess stroke related deficits for long period of time after stroke is a priority for the future use of animal models. For many of the tests in routine preclinical use, spontaneous recovery occurs rapidly after stroke, making long term studies challenging [11, 62–65]. However, understanding the profile of behavioural deficit and its recovery in different tests is important, as it will influence the interpretation of the effect of treatments on stroke outcome [65]. Whilst this study did not aim to recommend any specific test/s, we did want to determine the longevity of impairments and our ability to detect them after stroke.

Regular assessment of behavioural deficit in 104 animals for up to 24 weeks post stroke found rapid development of impairments specific to stroke animals. Whilst some deficits were acute and resolved rapidly, the basic behavioural test allowed discrimination between stroke and sham animals for the first week following stroke. Behavioural scales such as this have become popular tools for assessing stroke related deficits because they are simple to perform and require no specialised equipment, making them ideal for early assessment of stroke animals. Assessment scales such as the basic behavioural test used here may be akin to the NIHSS as they are most frequently used (and useful) during the early period after stroke. Being able to assess ischemia induced deficits quickly and easily early after the onset of MCAo is important. It allows screening of animals to assess the success of stroke induction, and offers the opportunity to randomise animals with equivalent neurological deficit into experimental treatment groups in equal proportions [26, 66].

Similar to other studies, we found the adhesive removal test effective in detecting long lasting deficits after stroke, with stroke animals significantly slower than shams to remove the contralateral tape for the entire experimental period [24, 25, 67–73]. However the ability of a test to detect behavioural deficit may in part depend on the location and size of the ischemic damage. For example, Trueman et al recently found no deficit in the adhesive removal test at 1 and 2 months post stroke [65]. The shorter occlusion duration of 30 minutes in Trueman’s study may have resulted in milder deficits and/or smaller infarct that did not involve the somatosensory cortex, or only mild damage that was amenable to recovery within 1 month.

The adhesive removal test is very relevant in linking experimental and clinical stroke, as it was originally based on the double simultaneous stimulation examination of contralateral neglect [74, 75]. Furthermore, it has been used across multiple species, namely mice, rats, gerbils and marmosets [20, 23, 24, 76, 77].

### Consistency in ischemic damage

Histological assessment of infarct volume remains the most frequently reported outcome in experimental stroke studies. This has followed the belief that reduction in infarct size is an indication that a therapy is neuroprotective, and will improve outcome for patients.

Evolution of ischemic damage after tMCAo occurs rapidly and as a function of time, involving four key stages: necrosis, inflammation, organisation, and resolution [78, 79]. Each stage follows in sequence: The infiltration of inflammatory cells is triggered by the initial necrosis. In turn, infiltrating macrophages facilitate clearance of necrotic tissue and formation of a fluid filled cavity. The rate of progression from neuronal necrosis to cavity formation was similar to that described by others [31, 37, 80]. However, this study adds to our understanding of the histological damage after stroke as it encompasses a greater number and range of timepoints (together with larger animal numbers) than previous studies (which have generally used only 3–4 animals per timepoint) [30, 31, 37, 79–81]. The randomised and blinded induction of ischemia and assessment of damage further enhance the value of the data presented here.

As the cellular and connective tissue makeup of the damaged area changes with progression of the infarct, this study highlights how the way in which damage is expressed becomes an important factor in order to allow comparisons across groups collected at different times post stroke. When the effects of oedema and atrophy were removed to allow expression of damage as one of total ischemic damage (as a proportion of contralateral hemisphere), all groups showed a similar proportion of damage. Together with similar CBF drop at MCAo and acute behavioural deficits, this suggests that there was good consistency in the area to experience ischemia, with this area going on to infarction, resolution and cavity formation. This highlights that our model has consistently induced ischemia to the MCA supplied cortex and striatum. Moreover, it suggests that this measure may allow better comparison of damage across experiments by different groups and could be introduced as a core assessment technique.

Volumetric measurements of damage alone do not reflect the structural and cellular changes occurring within the ischemic brain. Therefore, both qualitative and quantitative assessments must be made in order to appreciate the type and extent of ischemic damage, especially where multiple timepoints are being considered [11].

### Selecting appropriate endpoints and outcome measures

Selection of an appropriate outcome method and the time at which to assess stroke animal models are important considerations in experimental design. Understanding the variability in different outcome measures over time post stroke ensures an appropriate number of animals are assigned to each cohort to provide robust results. Similarly, we need to power our experiments for the most relevant outcome measure, and be mindful that other secondary outcome measures may not be powered for appropriately.

Using the most common outcome, histological damage, sample size calculations for different endpoints (and degrees of effect) found group sizes up to 12 animals could detect large effects (50 and 75% reduction in ischemic damage) across all timepoints. However, when smaller and probably more realistic effect sizes were considered (10 and 25% reduction in ischemic damage), much larger cohort sizes were required. When behavioural outcomes (adhesive removal test) were considered the primary measure, cohort sizes were greater again, given the larger degree of variability in performance.

The question of what and when to assess animal models of stroke will depend on each experiment and the hypothesised effect of the treatment. Whilst changes in infarct volume have been the most commonly reported outcome, it may not be the most useful in finding a treatment that will translate for acute stroke patients. Considering behavioural outcomes as the primary assessment would require a larger number of animals, akin to a clinical trial. This exploratory exercise presents challenges in aligning animal models with clinical stroke. The number of animals estimated here far outnumber the cohort sizes of 6–12 animals generally used in experimental studies and would present a practical and logistical challenge for any individual laboratory to undertake for a single study [82–85]. However collaborative efforts between laboratories may make such approaches more feasible [3, 86–88]. The methodologies required for such an approach have been developed by the EU funded Multi-PART consortium (http://www.dcn.ed.ac.uk/multipart/about.html).

It is also likely that different models, species and strains will produce different results, and require different numbers of animals per group. For example, Rosell showed that across different strains of mouse, the number of animals required per group ranged from <10 to >100 depending on the test and time examined [89]. Similarly, models that induce smaller, more consistent infarcts such as Endothelin-1 would lead to a smaller number of animals in each cohort [83].

### Limitations

In this study we aimed to reduce variability in infarct size by inducing stroke of 90 minutes duration in 15 week old male SHR rats. In order to understand stroke biology better and improve translation, replication and extension of this study to include females, aged animals, and animals with co-morbidities are required as these factors will all impact the development of ischemic damage and the resulting behavioural deficit [64]. Understanding the impact of each of these factors (and in combination) are important goals for researchers.

We limited our study to three behavioural tests of which the adhesive removal test proved most useful consistent with its links with neglect in patients. However there are a vast range of tests worth exploring for long term deficits after stroke [63]. Ultimately a battery of behavioural tests seems likely to be required but will need to be validated at extended times post-stroke. Time and costs prevented such validation in this study. A recent detailed study by Trueman et al assessed a range of tests and their ability to detect deficits at 2 months after stroke in rats [65]. Differences between species, strains, models, and within model conditions all need to be considered carefully when deciding whether a behavioural test is appropriate for the study.

Assessment of histological damage was limited to H&E staining in this study. Further exploration of the histological damage induced by stroke and the long term consequences of it are warranted. In addition, histology only provides a snapshot of the ischemic brain at that given time. Imaging would be advantageous if available, as it would allow a profile of ischemic damage over time within individual animals.

To ensure a consistent post-stroke environment, all animals were housed individually. However this may have had a detrimental effect on both behaviour and histology, as social isolation has been associated with increased histological damage and mortality, together with delayed recovery, an enhanced neuroinflammatory response, oxidative stress and oedema [90–95].

While the results presented here are based on the thread occlusion model, the lessons can be extrapolated to any stroke model. Researchers need to be aware of the variability of different tests and assessment outcomes, and their ability to detect differences both between stroke and sham animals, and within groups of stroke animals using their model of choice.

## Conclusions

What, when and how we assess stroke in animal models need careful consideration in moving stroke research forward. Traditional outcome measures of histological ischemic damage showed the progression of damage from an ischemic infarct to fluid filled cavity. When the degree of oedema or atrophy was accounted for, all timepoints experienced a similar degree of ischemic damage. Using the adhesive removal test as an example, here we have shown that deficits can be detected in stroke animals for up to 6 months after stroke. However sample size calculations powering an experiment to detect differences between groups of stroke animals resulted in large cohort sizes that would in most cases, necessitate multi-centre studies. Whilst long-term studies require an investment of time, finances, and patience, chronic endpoints are important for examining the long-term effects of a treatment and the inherent plasticity of the brain and will ultimately draw tighter links between experimental and clinical stroke.
